# Influence of Oral Contraceptive Use on Adaptations to Resistance Training

**DOI:** 10.3389/fphys.2019.00824

**Published:** 2019-07-02

**Authors:** Line B. Dalgaard, Ulrik Dalgas, Jesper L. Andersen, Nicklas B. Rossen, Andreas Buch Møller, Hans Stødkilde-Jørgensen, Jens Otto Jørgensen, Vuokko Kovanen, Christian Couppé, Henning Langberg, Michael Kjær, Mette Hansen

**Affiliations:** ^1^Section of Sport Science, Department of Public Health, Aarhus University, Aarhus, Denmark; ^2^Department of Orthopedic Surgery M, Bispebjerg Hospital and Center for Healthy Aging, Institute of Sports Medicine, Faculty of Health Sciences, University of Copenhagen, Copenhagen, Denmark; ^3^Steno Diabetes Center Aarhus, Aarhus University Hospital, Aarhus, Denmark; ^4^Department of Endocrinology and Internal Medicine, Medical Research Laboratories, Aarhus University Hospital, Aarhus, Denmark; ^5^Center of Magnetic Resonance, Aarhus University Hospital, Skejby, Denmark; ^6^Faculty of Health Sciences, University of Jyväskylä, Jyväskylä, Finland; ^7^CopenRehab, Department of Public Health, Faculty of Health and Medical Sciences, University of Copenhagen, Copenhagen, Denmark

**Keywords:** exercise, estrogen, estradiol, women, muscle hypertrophy, tendon, muscle strength

## Abstract

**Introduction:** The majority of young women use oral contraceptives (OCs). Use of OCs has been associated with lower myofibrillar protein and tendon collagen synthesis rates, but it is unknown whether OCs will limit the adaptive response of myotendinous tissue to resistance training.

**Design and Methods:** Fourteen healthy untrained young regular OC users (24 ± 1 years, fat% 32 ± 1, 35 ± 2 ml⋅min^-1^⋅kg^-1^) and 14 NOC users (non-OC, controls) (24 ± 1 years, fat% 32 ± 2, 34 ± 2 ml⋅min^-1^⋅kg^-1^) performed a 10-week supervised lower extremity progressive resistance training program. Before and after the intervention biopsies from the vastus lateralis muscle and the patellar tendon were obtained. Muscle (quadriceps) and tendon cross-sectional area (CSA) was determined by magnetic resonance imaging (MRI) scans, and muscle fiber CSA was determined by histochemistry. Maximal isometric knee extension strength was assessed by dynamometry while 1 repetition maximum (RM) was determined during knee extension.

**Results:** Training enhanced CSA in both muscle (*p* < 0.001) and tendon (*p* < 0.01). A trend toward a greater increase in muscle CSA was observed for OC (11%) compared to NOC (8%) (interaction *p* = 0.06). Analysis of mean muscle fiber type CSA showed a trend toward an increase in type II muscle fiber area in both groups (*p* = 0.11, interaction *p* = 0.98), whereas type I muscle fiber CSA increased in the OC group (*n* = 9, 3821 ± 197 to 4490 ± 313 mm^2^, *p* < 0.05), but not in NOC (*n* = 7, 4020 ± 348 to 3777 ± 354 mm^2^, *p* = 0.40) (interaction *p* < 0.05). *Post hoc* analyses indicated that the effect of OCs on muscle mass increase was induced by the OC-users (*n* = 7), who used OCs containing 30 μg ethinyl estradiol (EE), whereas the response in users taking OCs with 20 μg EE (*n* = 7) did not differ from NOC. Both the OC and NOC group experienced an increase in maximal knee strength (*p* < 0.001) and 1RM leg extension (*p* < 0.001) after the training period with no difference between groups.

**Conclusion:** Use of OCs during a 10-week supervised progressive resistance training program was associated with a trend toward a greater increase in muscle mass and a significantly greater increase in type I muscle fiber area compared to controls. Yet, use of OCs did not influence the overall increase in muscle strength related to training.

## Introduction

Worldwide, more than 100 million fertile young women use oral contraceptives (OCs) for menstrual regulation and contraception (Christin-Maitre, 2013). It has been suggested that OCs may affect sports performance through influence on steroid hormone receptors located in the peripheral tissues such as skeletal muscle and tendon (Liu et al., 1996; Wiik et al., 2005; Ekenros et al., 2017). Yet, knowledge on the effects of OC on long term adaptations to training is sparse.

Most women use monophasic OC pill formulations containing synthetic estrogen, ethinyl estradiol (EE) and synthetic progesterone, progestin, in a constant dose during the pill cycle. Depending on brand name and OC “generation,” the formulation of EE typically varies between 20 and 50 μg daily dose, while the different types of progestins differ in dose depending on potency (Stanczyk, 2003; Burrows and Peters, 2007). In addition, progestins also differ in androgenocity introducing a potential influence on adaptations to training (Ruzic et al., 2003). Furthermore, use of OCs is associated with reduced endogenous levels of natural estrogen, progesterone, free testosterone and insulin-like growth factor I (IGF-I), but enhanced levels of cortisol (Hansen et al., 2011; Zimmerman et al., 2014; Eisenhofer et al., 2017), which may also affect the anabolic response to training.

To study the effects of OC on exercise induced muscle growth, resistance training seems as the optimal exercise modality because of its well-known hypertrophic effects (Folland and Williams, 2007). Nonetheless, only a few studies have so far investigated the potential role of OCs in relation to adaptation to resistance training (Ruzic et al., 2003; Nichols et al., 2008). One study showed no difference in strength gain between users (*n* = 13) and non-users of OC (*n* = 18) following 12 weeks of resistance training (Nichols et al., 2008), but no information on OC type was provided. This may be of importance since a smaller strength gain has been reported following 16 weeks resistance training in users of anti-androgenic OCs (EE and cyproterone acetate, *n* = 26) compared to users of high androgenic OCs (EE and levonorgestrel, *n* = 24) (Ruzic et al., 2003). Unfortunately, no control group of non-OC users was included in the latter trial, making the interpretation of the results challenging.

Changes in skeletal muscle mass are determined by the balance between myofibrillar protein synthesis and breakdown. In users of low androgenic OCs (EE and gestoden) myofibrillar fractional protein synthesis rate has been reported to be lower 24 h following one-legged kicking exercise compared to non-users of OC (Hansen et al., 2011). The influence of OCs on myofibrillar protein breakdown rate has not been clearly elucidated, and has only been studied indirectly by analyzing interstitial fluid obtained from the skeletal muscle for 3-methyl-histidine, which did not show a difference between OC users and non-users (Hansen et al., 2011). Based on the sparse existing data, we therefore hypothesized, that OC use may negatively influence the gain in skeletal muscle mass and strength in response to resistance training compared to non-users of OC, but the effect may depend on the type of OC (content of EE and type of progestin).

The biomechanical properties of tendon and ligaments improve in response to regular training (Svensson et al., 1985), and regular resistance training has been shown to induce tendon hypertrophy in groups of men (Kongsgaard et al., 2007) and in a mixed group of men and women (Arampatzis et al., 2007). However, cross-sectional data in runners suggests that the ability to adapt to training is reduced in women (Westh et al., 2008). Since tendon adaptations have important perspectives related to lowering of the risk of injury in tendon and ligaments, the effect of regular resistance training as a function of OC use must be clarified. It has been suggested that circulating estrogen (endogenous or exogenous) alters the risk of injuries by changing the structural composition and adaptations to exercise, and thereby also the mechanical properties of the tendon (Magnusson et al., 2007; Hansen and Kjaer, 2016). In support, estradiol increases tendon collagen synthesis and the overall tendon collagen turnover, while it reduces tendon and ligament stiffness (Lee et al., 2015). In contrast, use of OCs containing EE is associated with a lower tendon collagen synthesis rate in the patellar tendon compared to non-users both at rest and after acute knee-extensor exercise (Hansen et al., 2008, 2009a). Yet, no previous studies have looked into how OC use influences the response to regular resistance training.

The overarching aim of the study was therefore to compare adaptations in skeletal muscle and tendon to 10 weeks of resistance training in users of low-androgenic OCs vs. NOC. We hypothesized that OC use reduces the anabolic effect of resistance training in the skeletal muscle and tendon.

## Materials and Methods

### Subjects

Thirty young, healthy women already using 3rd generation OCs or using no OCs were recruited through local newspapers and included in the study. All participants were non-smokers, non-users of medication, and without orthopedic and metabolic disorders that would influence their participation in the training intervention. Exclusion criteria were: resistance training more than once every month within the last 6 months, other types of physical training more than 2 h per week, bike transportation more than 20 km per day, and being on a hypocaloric diet to reduce weight.

The 14 OC users had used OCs for 6.1 ± 5.0 years. Third generation OCs are characterized as being low-androgenic (Burrows and Peters, 2007). The brand names of the OCs were Lindynette (30 μg EE and 75 μg gestoden per day, *n* = 7), Gestonette (20 μg EE and 75 μg gestoden per day, *n* = 5), or Novynette (20 μg EE and 150 μg desogestrel per day, *n* = 2). The 14 NOCs had regular menstrual cycles for at least 1 year (within the range of 24–35 days). The subjects were informed about the risks and benefits associated with the study and provided written informed consent. The protocol followed the Declaration of Helsinki and was approved by the Central Denmark Region Committees on Health Research Ethics (Journal No.: M-20100187).

### Design

**FIGURE 1:**
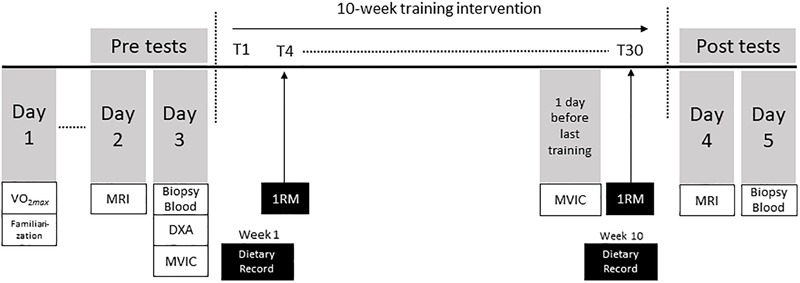
Study overview.

The study compared adaptations in skeletal muscle and tendon after 10 weeks of resistance training in OC and NOC users (Figure 1). Each participant visited the laboratory on three separate occasions within 1–2 weeks prior to the start of training period (baseline). At the first visit height, weight, body composition [Dual-Energy x-ray absorptiometry (DXA)], aerobic fitness (VO_2max_/kg) determined indirectly by a submaximal bike test (Åstrand and Rodahl, 1986), and physical activity (MET/24 h) level determined by questionnaire (Aadahl and Jorgensen, 2003). At the same day, the participants were familiarized with the testing procedures of the 1 repetition maximum (RM) and isometric dynamometer measurements to minimize any learning effects. At the second visit magnetic resonance imaging (MRI) of the thigh muscle and patellar tendon were collected. MRI was performed on average 5.9 days [range 2–13 days] after the initial visit and before collection of strength measures and tissue samples to avoid edema. At the third visit, blood, tendon and muscle tissue samples were collected in the morning after an overnight fast, and isometric muscle strength was determined by dynamometry. Muscle and tendon samples were collected in either the dominant or non-dominant leg as determined by randomization. The isometric muscle strength was determined in the leg that had not been biopsied. All tests were repeated after the resistance training intervention period (post-training). The isometric dynamometer strength was performed on the day before the last training session. MRI data were collected before the blood, muscle and tendon samples, at 48 or 72 h after the last training session. In addition, 4 days of self-reported dietary intake were recorded in week 1 and 10 of the intervention period (Vitakost, Kolding, Denmark) and recreational physical activity logs were collected during the training intervention. The participants were asked to remain weight-stable during the intervention.

### Resistance Training Protocol

Test and training sessions were completed at the Section for Sports Science at Aarhus University. The exercise protocol consisted of 10 weeks of progressive resistance training performed 3 times per week supervised by physical therapists. Training intensities were estimated from the 1 RM test. The exercises consisted of seated knee extensions and inclined leg press performed in a progressive manner; week 1: 3x 12 repetitions of 15 RM; week 2–3: 3x 12 repetitions of 12 RM; week 4–5: 3x 10 repetitions of 10 RM; week 6–10: 4x 10 repetitions of 10 RM. Each training session was initiated with a 10 min warm-up on a stationary bike. The 1 RM test of knee extension strength was performed at the fourth training session (baseline test), and was repeated at every third training session as well as during the last training session of the intervention period (post-test). All subjects were provided with a standardized amount of protein (Maxim Pink protein bar, 10.6 g protein) after each training session.

### 1RM Strength Test

Participants warmed-up with 10 min ergometer cycling (60 W) followed by six repetitions at 60% of the 1 RM predicted from the familiarization session. The estimated starting weight was 85% of the previous 1 RM. The load was increased or reduced until a true 1 RM using a full range of motion was reached. Three to five trials separated by 5 min rest was allowed. One participant from each group was unable to perform the 1RM test after the training, reducing n to 13 in each group.

### Isometric Dynamometer Strength

Participants were seated in an isometric dynamometer (Humac Norm, CSMi, Stoughton, MA, United States) with a hip angle of 90° and strapped by the hip. The non-working leg was positioned behind a stabilizing bar and participants were asked to keep their arms crossed over the chest. The rotational axis of the dynamometer was aligned with the transverse knee-joint axis of the working leg and attached to the lower leg by a length adjustable lever arm 3 cm proximal to malleolus medialis. After a standardized instruction (to contract as strongly and fast as possible) and two familiarization attempts, the subjects performed three to five (depending on the consistency of the first trials) maximal voluntary isometric contractions (MVIC) for the knee extensors at a knee angle of 70°. The subjects received visual feedback and verbal encouragement during the maximal contractions. All contractions were separated by at least 1 min. of rest. The attempt with the highest MVIC, defined as peak torque measurement, was used for further analysis. All isometric strength data were sampled and exported using TeleMyo Direct Transmission System and MyoResearch Software (Noraxon USA, Scottsdale, AZ, United States). A sampling frequency of 1500 Hz was applied and the final analyses were performed using custom-made software. To normalize data, all strength measurements were scaled to body-weight (Jaric, 2003; Aagaard et al., 2007).

### Muscle and Tendon Cross-Sectional Area (CSA)

Magnetic resonance imaging of muscle and tendon CSA was performed at the MR Centre at Aarhus University Hospital, Aarhus, Denmark. No strenuous exercise was performed within 48 h before the baseline-MRI and the post-MRI was performed 48–72 h after the last training session. This was done to minimize the risk of an exercise induced increase in muscle water content (Kristiansen et al., 2014). Participants were placed in a supine position with fully extended, relaxed legs. Muscle CSA was measured 20 cm proximal to the tibia plateau (TP), using MR-images (Siemens Magnetom Avanto 1.5 Tesla, Siemens AG, Munich, Germany, T1 weighted) from two lower extremity coils. An initial image was used to determine the proximal, lateral plateau of tibia and this point was then used as the reference position. The muscle CSA of vastus lateralis was manually outlined using the software program OsiriX DICOM Viewer (version 7, OsiriX HD, 2011 Pixmeo SARL, Bernex, Switzerland).

The Patellar tendon CSA was measured in the same scanner in three anatomical regions directly beneath the patellar insertion (proximal) by determining the first picture on which the patella was not present, just above the tibia insertion (distal) by determining the first picture on which Hoffa’s fat pat was no longer seen, and midway between these two regions (central), using MR-images with the following parameters: repetition time (TR)/echo time (TE) 400/15 ms, field of view (FOV) 160, matrix 256 × 256, slice thickness 3 mm (Hansen et al., 2013). The MRIs of the Patellar tendon were performed using a special knee coil placing the knee in a fixed angle of ∼120°. The perpendicular angle of the tendon was set using a localizer, and axial tendon slices were obtained. Patellar tendon CSA was manually outlined using the software program OSIRIX 7.

For both muscle and tendon data analyses were performed by a single technician in a blinded fashion. A mean value of three measurements of the same image was calculated and used for analysis (Supplementary Figure S1). The coefficient of variation was found to be less than 5%. One participant from the NOC group did not have an MRI scan due to claustrophobia.

### Collection and Analyses of Muscle Tissue

Participants were asked to avoid physical activity 48 h before biopsy collection. Muscle tissue samples were collected from *m. vastus lateralis* of the dominant or the non-dominant leg determined in a random fashion using a 5 mm Bergström needle under local anesthetic (1% lidocaine) after an overnight fast. Muscle samples of ∼100 mg were dissected free of fat and connective tissue, and one part was embedded in TissueTek and then frozen in pre-cooled isopentane, and the other part was snap frozen in liquid nitrogen. All samples were stored at -80°C until analysis.

Standard ATPase staining performed on 7 μm serial sections was used to evaluate fiber type distribution and muscle fiber CSA (Brooke and Kaiser, 1970). The serial sections were visualized and analyzed using a Leica DM2000 microscope (Leica, Stockholm, Sweden) and a Leica Hi-resolution Color DFC camera (Leica, Stockholm, Sweden) combined with image-analysis software (Leica Qwin ver. 3, Leica, Stockholm, Sweden) essentially as previously described (Dalgas et al., 2010). Briefly, a fiber mask was automatically drawn by the software based on a immunohistochemically staining of the basement membrane. This mask was fitted manually to the cell borders of the included muscle fibers. Only fibers cut perpendicularly to their longitudinal axis were used for the determination of fiber size. Images of the three ATPase stainings were then fitted with the fiber mask. Descriptive statistical analysis by the software allowed determination of the relative proportion of the various fiber types and fiber CSA. Blinded analyses were performed by trained laboratory personnel. The determination of muscle fiber CSA and typing of fibers were carried out essentially in accordance with the procedure described by Andersen et al. (Andersen and Aagaard, 2000). Calculation of muscle fiber distribution was performed for the three fiber types (I, IIa, and IIx) and muscle fiber CSA was performed for the two major fiber types (I and II). For the analysis of muscle fiber type distribution, *n* = 13 from each group before and after the intervention period (average number of fiber per subject 148 ± 3). Several of the muscle biopsy slides for histochemical analysis were destroyed due to freeze damage and problems with the analysis procedure. Therefore, the analysis of changes in muscle fiber area, was performed on *n* = 9 for OC and *n* = 7 for NOC (average number of fibers per subject 123 ± 6) and type IIx muscle fibers were not included in the analysis due to absence or very low numbers particularly at the post-time-point.

### Muscle Protein Expression

Western blot analyses were used to assess protein expression and phosphorylation levels of muscle proteins. Muscle samples were homogenized in an ice cold buffer containing (in mM) 50 HEPES, 137 NaCl, 10 Na_4_P_2_O_7_, 10 NaF, 2 EDTA, 1 MgCl_2_, 1 CaCl_2_, 2 Na_3_VO_4_, 1% (vol/vol) Nonidet P-40, 10% (vol/vol) glycerol, 2 μg/ml aprotinin, 5 μg/ml leupeptin, 0.5 μg/ml pepstatin, 10 μg/ml antipain, 1.5 mg/ml benzamidine, and 100 μM 4-(-2-aminoethyl)-benzenesulfonyl fluoride, hydrochloride (pH 7.4), and rotated for 60 min at 4°C. Subsequently, the samples were centrifugation at 14,000 × *g* for 20 min at 4°C and the pellet discharged. With this method, we have previously been able to detect many skeletal muscle proteins, however, we might also lose proteins of interest (Murphy and Lamb, 2013). Protein concentration in the supernatant was determined using the Bradford assay. After separating the proteins by gel electrophoresis and blotting them on to a PVDF membrane, the membranes were incubated overnight with the following antibodies: from Cell Signaling Technology (MA, United States): mTOR (cat. no. 2972), p-mTOR Ser^2448^ (cat. no. 2971), p70S6K (cat. no. 9202), eIF4E (cat. no. 9742), AKT-pan (cat. no. 4691), p-AKT Ser^473^ (cat. no. 9271), 4EBP1 (cat. no. 9644), non-p-4EBP1 Thr^46^ (cat. no. 4923), and LC3B (cat. no. 3868); from Abcam (Cambridge, United Kingdom): Fbx32 (cat. no. ab92281) and MURF1 (cat. no. ab172479). Horseradish peroxidase (HRP)-conjugated goat anti-rabbit or goat anti-mouse IgG were used as secondary antibodies and visualized by BioWest enhanced chemiluminescence (Pierce). Quantification was performed using UVP Bio Imaging System software (UVP, Upland, CA, United States). Expression of both non-phosphoprotein and phospho-protein are presented as ratios of target protein to total protein load quantified using the Stain Free Technology. Due to difficulties in obtaining enough muscle tissue, the analysis was performed on *n* = 8 for OC and *n* = 12 for NOC.

### Tendon Samples

Tendon biopsies were randomly obtained from the dominant or the non-dominant leg, according to procedures described in detail elsewhere (Hansen et al., 2009c; Kongsgaard et al., 2010). In brief, following sterilization of the insertion site, the skin was injected with local anesthetic (1% lidocaine), and a 3- to 5-mm-long incision was made distally to the patella apex. A tendon biopsy was obtained by using a 16-G Monopty biopsy instrument (Bard, Covington, GA, United States) with a disposable core biopsy needle (14 gauge). The biopsy needle was inserted into the tendon surface at a ∼30° angle and fired, securing a tissue sample of approximately 8–10 mg. The tissue sample was cleared of external adipose tissue and blood, snap-frozen in liquid nitrogen and stored at -80°C for subsequent analysis.

Biochemical analysis of collagen cross-links was performed as previously described in details (Hansen et al., 2013).

Freeze-dried tendon samples were hydrolyzed and re-dissolved in H_2_O. The collagen-specific cross-linking compounds hydroxylysyl pyridinoline (HP), lysyl pyridinoline (LyP), and pentosidine were analyzed spectrophotometrically by reversed-phase high-performance liquid chromatography (HPLC) to quantify collagen protein (Creemers et al., 1997).

### Analyses of Blood Parameters

All blood samples were taken in the morning after an overnight fast (<6 h) and from an antecubital vein. Serum albumin, estradiol, progesterone, testosterone, and sex hormone binding globin (SHBG) were determined at baseline and after the training intervention (post) by standard procedures performed at the Department of Clinical Biochemistry at the Aarhus University Hospital. For each variable, all samples were measured in the same assay run.

### Statistical Analyses

All data are reported as means ± SE unless stated otherwise. All data were tested for normal distribution by visual inspection and using a Shapiro-Wilk normality test. Statistical analyses for differences between OC and NOC in subject characteristics, serum hormones levels, fibril characteristics and tendon content of collagen and cross-links were performed using an unpaired *t*-test. A paired *t*-test was used to test for differences between baseline and post-in serum albumin, progesterone and testosterone. The log-transformed normal distributed data for FM, weight, PAL and BMI was used in an unpaired *t*-test since the data did not pass the normality test. Estradiol and SHBG did not pass normality tests even after log-transformation, thus a Wilcoxon Signed-Rank test was used to test for significance in the raw data between OC and NOC.

Test for difference in muscle CSA, muscle fiber CSA, muscle strength, tendon CSA and tendon cross-links caused by training load or use of OC was performed using a two-way ANOVA with repeated measurements followed by a Holm-Sidak *post hoc* test. Data for tendon CSA at the mid and distal level only passed the normality test after log-transformation. Therefore, log-transformed data for these parameters were used in the analysis. Unpaired *t*-tests were performed to test for differences in response to training in muscle CSA between different types of OC (containing either 20 or 30 μg EE/pill) and NOC. A Spearman correlation was used to test the association between daily protein intake per kg bodyweight and change in muscle CSA after the intervention. The level of significance was set at *p* ≤ 0.05, while *p* ≤ 0.10 was designated as a trend. The following labels were used: ^∗∗∗^*p* < 0.001, ^∗∗^*p* < 0.01, and ^∗^*p* ≤ 0.05 unless stated otherwise. The statistical analyses were performed using the statistical software package Sigma Plot version 13.0.

## Results

The OC and NOC groups were comparable regarding age, height, weight, body mass index (BMI), physical activity level (MET/24 h), aerobic fitness (VO_2max_/kg), and body composition (Table 1). The recreational activity logs were checked for changes in activity level during the intervention period, but none of the participants performed strenuous physical training more than 2 h per week and no additional resistance training was performed besides the intervention. Every one of the included participants completed the study. All participants followed and received individual supervision for each training session, and compliance with the training protocol was 100%, as any missed training session was performed another day within the same week.

**Table 1:** Subject characteristics.

	NOC (*n* = 14)	OC (*n* = 14)
Age (years)	24 ± 1	24 ± 1
Height (m)	1.67 ± 0.02	1.68 ± 0.02
Weight (kg) (Baseline)	66.7 ± 2.1	65.6 ± 3.6
Weight (kg) (Post)	67.3 ± 2.1	66.6 ± 4.1
BMI (kg⋅m^-2^)	24 ± 1	23 ± 2
Body Fat (%)	32 ± 2	32 y2
Aerobic fitness, VO_2max_ (ml⋅min^-1^⋅kg^-1^)	32 ± 2	35 y3
Physical activity level (MET/24 h)	1.8 ± 0.1	1.6 ± 0.1
Use of OC (years)	0 ± 0	6 ± 1***

*BMI, body mass index; Aerobic fitness, maximal oxidative uptake of oxygen/min/kg body weight; OC, oral contraceptives; NOC, non-users of oral contraceptives. No significant differences existed between groups except for use of OC (^∗∗∗^ denotes *p* < 0.001). Values are mean ± SE.*

Fourteen OC and 14 NOC users were eligible to be included in the analysis. Two NOC were excluded before the analysis, since they had deliberately reduced their energy intake in the intervention period and lost 8.4 and 7.3% in body weight, respectively, which may have reduced the anabolic potential of resistance training on muscle growth.

### Muscle CSA and Muscle Fiber CSA

**FIGURE 2:**
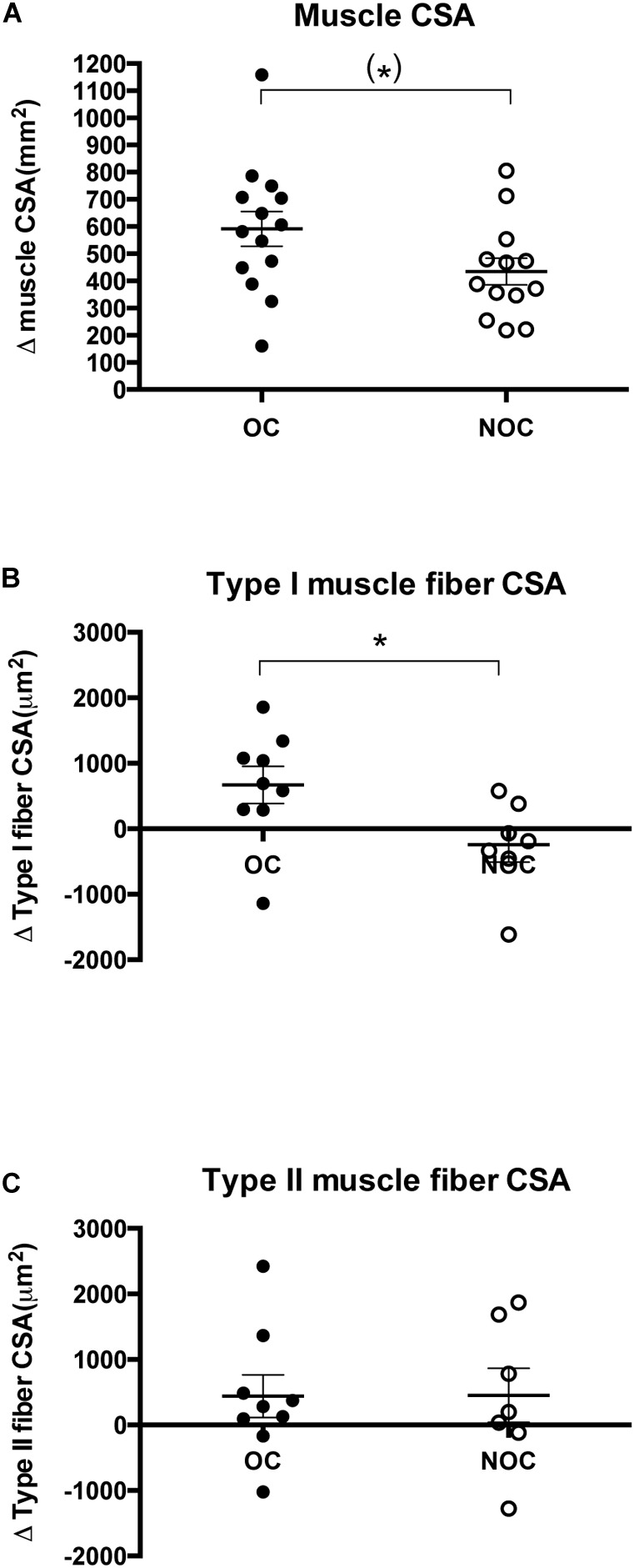
Changes in muscle CSA and muscle fiber CSA. **(A)** Change in muscle cross-sectional area (mm^2^) in OC and NOC users (*n* = 14 and *n* = 13, respectively) from baseline to post-training. Change in cross-sectional area (μm^2^) of type I **(B)** and type II **(C)** muscle fibers in the OC and NOC group (*n* = 9 and *n* = 7, respectively) from baseline to post-training. OC is illustrated by closed circles, while NOC are shown by open circles. The horizontal line denotes mean ± SE. (^∗^) denotes a trend (*p* = 0.66), while a ^∗^ denotes *p* < 0.05.

Resistance training induced an increase in muscle CSA in both OC (10.8 ± 1.3%, *p* < 0.001) and NOC users (7.9 ± 0.09%, *p* < 0.001) (Figure 2A and Supplementary Figure S1). Furthermore, a trend toward interaction between OC status and training effect was observed (*p* = 0.06), which indicated a greater increase in muscle CSA in the OC group than in the NOC group. OC sub analysis indicated that the increased muscle mass was driven by the OC-users (*n* = 7), who used OCs containing 30 μg EE, whereas the response in users taking OCs with 20 μg EE (*n* = 7) did not differ from NOC (30 vs. 20 μg EE: 13.1 ± 1.7% vs. 8.5 ± 1.4%, *p* = 0.08. 30 μg EE vs. NOC: *p* = 0.01. 20 μg EE vs. NOC: *p* = 0.73) (Supplementary Figure S2).

For type I muscle fiber CSA, the two-way ANOVA demonstrated a significant interaction between OC status and time (*p* = 0.04) (Figure 2B). In OC users, type I muscle fiber CSA was significantly greater after the training period (3821 ± 197 to 4490 ± 313 μm^2^, *p* < 0.05), whereas no significant change was observed in NOC users (NOC: 4020 ± 348 to 3777 ± 354 μm^2^, *p* = 0.40). Type II muscle fiber CSA showed a non-significant increase after the training period (*p* = 0.11) in both groups (OC: 3452 ± 242 to 3891 ± 387 μm2, NOC: 3239 ± 344 to 3691 ± 361 μm^2^), but no interaction between OC status and time was observed (*p* = 0.98) (Figure 2C).

The training intervention induced a change in muscle fiber type distribution (Table 2). The training intervention reduced the relative number of type IIx muscle fibers (*p* < 0.001), while the relative type IIa muscle fiber number increased (*p* < 0.05). The changes in type IIx muscle fiber number (*p* = 0.05) was greater in OC than NOC. In contrast, type I muscle fiber relative number did not significantly change after the intervention.

**Table 2:** Muscle fiber type distribution.

	OC (*n* = 13)		NOC (*n* = 13)		Two-way ANOVA RM		
Fiber type%	Baseline	Post	Baseline	Post	Training	Group	Interaction
Type I	46.9 ± 2.8	48.3 ± 2.5	53.4 ± 2.7	52.4 ± 2.9	0.91	0.13	0.52
Type IIa	42.6 ± 2.5	47.7 ± 2.5	39.4 ± 2.6	42.8 ± 2.1	<0.05	0.16	0.64
Type IIx	10.5 ± 2.2	3.9 ± 1.5	7.1 ± 2.1	4.8 ± 1.2	<0.001	0.59	0.05

*The relative distribution (%) of type I, type IIa, and type IIx muscle fibers in OC users and NOC users is shown. Data are reported as mean ± SE.*

### Muscle Strength

**FIGURE 3:**
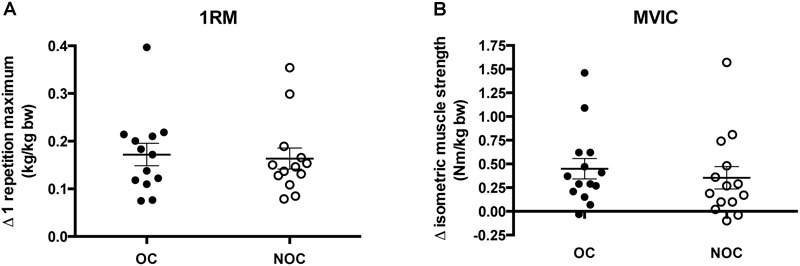
Changes in muscle strength. **(A)** Difference in 1 repetition maximum (1RM) in OC and NOC users from baseline to post-training (kg) (*n* = 13 in each group). **(B)** Changes in maximum voluntary isometric strength (MVIC) in users of OC and NOC users from baseline to post-training (Nm/Kg Bw) (*n* = 14 in each group). Bw, body weight; OC, closed circles; NOC, open circles. The horizontal line denotes mean ± SE.

Absolute muscle strength increased in both OC and NOC users following the training intervention as measured by 1RM test (OC: 18.2 ± 3.6% vs. NOC: 15.4 ± 1.6%; time *p* < 0.001) and MVIC (OC: 17.3 ± 3.6% vs. NOC: 13.8 ± 3.5%; time *p* < 0.001). Larger absolute increases were observed in the OC users, however, no significant difference between the two groups was observed in 1RM (interaction *p* = 0.46) and MVIC (interaction *p* = 0.36). Normalizing data to individual baseline and post-body weight (bw) did not influence the results (1RM; OC: 1.12 ± 0.05 kg/kg bw (baseline) vs. 1.29 ± 0.05 kg/kg bw (post); NOC: 1.05 ± 0.09 kg/kg bw (baseline) vs. 1.20 ± 0.10 kg/kg bw (post), *p* < 0.001 for training effect, interaction *p* = 0.66. MVIC; OC: 3.0 ± 0.14 Nm/kg bw (baseline) vs. 3.5 ± 0.18 Nm/kg bw (post); NOC: 3.0 ± 0.17 Nm/kg (baseline) vs. 3.3 ± 0.19 Nm/kg (post), *p* = 0.01 for training effect, interaction *p* = 0.56) (Figure 3).

### Muscle Tissue Protein Expression

The mTOR expression increased with time (*p* < 0.05), while Murf-1 showed a trend toward a decrease at the post-time point (*p* = 0.08). eIF4E and p70S6K showed a positive interaction between time and OC-status (*p* < 0.05) revealing an decreased expression in OC users and an increased expression in NOC users after the training period. In contrast, protein expression of mTOR^Ser2448^, Akt2, Akt^Ser473^, 4EBP1, 4EBP1^Thr46(non-p)^, Atrogin-1, and the ratio of LC3B-II/LC3B-I were not different between OC and NOC groups (Supplementary Figure S3).

### Tendon Cross Sectional Area and Cross-Links

Tendon CSA (proximal, mid, and distal) did not differ between the dominant and non-dominant leg and only average values for both legs are shown in Table 3. After the training period, tendon CSA at all three levels (proximal, mid, and distal) was significantly increased compared to baseline. However, no difference between groups was observed in the response to training.

**Table 3:** Tendon CSA, and concentrations of collagen and collagen cross-links.

	OC (*n* = 14)		NOC (*n* = 14)		Two-way ANOVA RM		
	Baseline	Post	Baseline	Post	Training	Group	Interaction
Tendon CSA proximal	81 ± 6	87 ± 5	77 ± 3	85 ± 5	<0.05	0.71	0.70
Tendon CSA middle	78 ± 4	90 ± 4	80 ± 3	97 ± 7	<0.001	0.37	0.57
Tendon CSA distal	95 ± 5	101 ± 5	100 ± 5	109 ± 5	<0.05	0.31	0.57

	**OC (*n* = 14)**		**NOC (*n* = 11)**				
	**Baseline**	**Post**	**Baseline**	**Post**	**Training**	**Group**	**Interaction**

Collagen	0.62 ± 0.02	0.64 ± 0.02	0.61 ± 0.03	0.62 ± 0.04	0.61	0.66	0.72
HP/Collagen	0.73 ± 0.06	0.80 ± 0.05	0.63 ± 0.06	0.65 ± 0.06	0.39	0.07	0.56
LyP/Collagen	0.03 ± 0.02	0.03 ± 0.00	0.04 ± 0.01	0.03 ± 0.00	0.18	0.81	0.13
Pentosidine/Collagen	0.012 ± 0.001	0.012 ± 0.001	0.011 ± 0.001	0.012 ± 0.001	0.47	0.46	0.44

*CSA (mm^*2*^) at the proximal, middle and distal part of the patella tendon in OC users and NOC users is reported along with collagen concentration (mg/mg d.w; dry weight) and collagen cross-links of tendon as fractions of HP, LyP, and pentosidine relative to collagen (pmol/pmol). Data are shown as mean ± SE.*

Analysis of tendon collagen content (dry weight), and cross-links (fractions of HP, LyP, and pentosidine) was performed in 14 OCs and 11 NOCs, since attempts to obtain a tendon biopsy were not successful in three NOCs (Table 3). A trend for higher concentration of HP in the OC than the NOC group was observed (*p* = 0.07), but otherwise no group difference between OC and NOC or difference in response to training were observed (Table 3).

### Blood Parameters

At baseline and post-training, serum (s-) albumin and s-SHBG were higher in OC users compared to NOC, whereas s-estradiol, s-progesterone, and s-testosterone were lower in the OC compared to the NOC group (Table 4). S-estradiol was below the detection limit in 11 out of 14 OC-users at baseline and 8 out of 14 OC users after the intervention. The SHBG level in NOC users was higher post-training than at baseline (*p* < 0.001). Similarly, NOC mean serum progesterone tended to be higher after the intervention vs. baseline (*p* = 0.06).

**Table 4:** Blood parameters.

		NOC (*n* = 14)	OC (*n* = 14)	*P*-values
Albumin (g/L)	*Baseline*	42 ± 1	46 ± 1	<0.01
	*Post*	42 ± 1	46 ± 1	<0.05
Estradiol (nmol/L)	*Baseline*	0.45 ± 0.06	11 out of 14 < 0.05 nmol/L	<0.05
	*Post*	0.72 ± 0.17	8 out of 14 < 0.05 nmol/L	<0.05
Progesterone (nmol/L)	*Baseline*	15.2 ± 4.2	1.1 ± 0.1^a^	<0.01
	*Post*	27.2 ± 8.8	1.1 ± 0.2^b^	<0.05
Testosterone (nmol/L)	*Baseline*	1.18 ± 0.14	0.76 ± 0.07	<0.05
	*Post*	1.35 ± 0.12	0.90 ± 0.09	<0.05
SHBG (nmol/L)	*Baseline*	57 ± 6	204 ± 14	<0.01
	*Post*	65 ± 7^#^	224 ± 13	<0.01

*Serum concentrations of albumin, estradiol, progesterone, testosterone, and sex hormone binding globin (SHBG) in OC and NOC at baseline and post-training is reported. Data are shown as mean ± SE. ^*a*^2 out of 14 in OC had a progesterone level < 0.03 nmol/L. ^*b*^One out of 14 in the OC group had a progesterone level <0.03 nmol/L. A ^#^ designate a significant difference between baseline and post-training values, *p* < 0.05. *P*-values designate differences between OC and NOC.*

### Energy and Macronutrient Intakes

Eating habits were not different at week 1 and 10 of the intervention. Therefore, only average energy and macronutrient intake from the two periods are included in Table 5. One subject from each group was excluded from the analysis due to unreliable energy intake registration based on cut-off values (Goldberg et al., 1991), despite that all subjects were weight-stable during the intervention. No significant difference in energy intake, fat, carbohydrate and protein intake was observed between groups, but the protein intake per kg body mass was higher in OC than NOC users.

**Table 5:** Energy and macronutrient intake.

	OC (*n* = 13)	NOC (*n* = 13)	*P-*values
Energy intake (kJ/day)	8390 ± 1960	8630 ± 1716	0.74
Carbohydrates, E%	52 ± 4	49 ± 4	0.07
Fat, E%	27 ± 4	30 ± 6	0.17
Protein, E%	17 ± 4	15 ± 2	0.06
Protein, g/day	81 ± 10	74 ± 14	0.15
Protein g/kg bw/day	1.3 ± 0.2	1.1 ± 0.2	0.03

*The average of two 4-day self-reported food records performed in week 1 and 10 of the intervention is shown for OC and NOC. E%, % of total daily energy intake; Bw, body weight. Data are shown as mean ± SE.*

## Discussion

Studies investigating the influence of OCs on adaptation to resistance training are very few and conflicting. Here, we present the first data based on a controlled intervention focused on elucidating the effect of a single type of OC (3rd generation). Furthermore, the participants performed one-on-one supervised resistance training with a high level of compliance and a controlled progressive overload. These factors are important when interpreting the results since other studies have used a mix of OCs (Ruzic et al., 2003; Nichols et al., 2008; Ekenros et al., 2013; Wikstrom-Frisen et al., 2017) and failed to improve muscle strength following the resistance training intervention probably due to a less controlled (and intense) training intervention (Wikstrom-Frisen et al., 2017).

Contrasting to our hypothesis, a trend toward a larger increase in muscle mass (MRI) (*p* = 0.06) was observed in OC compared to NOC users (11 vs. 8%). This contrasts our expectations based previous data showing a lower myofibrillar fractional protein synthesis rate in OC users of a similar type of low androgenic OC (EE and gestoden) compared to NOC (Hansen et al., 2011). Previous exercise studies in the field have failed to show any clear effect of OC on adaptation to resistance training. For example, one study by Nichols et al. (2008) showed no difference between OC and NOC in muscle strength gain after a 12-week strength period; however, included users of different types of OCs and included different types of athletes, who continued their discipline-specific training during the intervention (Nichols et al., 2008). These factors may have introduced bias and thereby limited the possibility of detecting group differences. The type of OC may influence the response to training as reported by Ruzic et al. (2003), who compared the effect of 16-weeks of resistance training in users of OCs containing either EE and the anti-androgen cyproterone acetate or EE and levonorgestrel (Ruzic et al., 2003). The latter group showed an increased gain in fat-free mass and muscle strength after the intervention compared to the first group, indicating either an anti-anabolic effect on skeletal muscle of the anti-androgen OCs or a stimulating effect of the OC containing levonorgestrel. The lack of a NOC control group leaves this comparison unresolved, but the data underline the need for elucidating the isolated effect of the different types of OCs on training adaptabilities.

Muscle hypertrophy is experienced when the average myofibrillar protein synthesis rate over time exceeds the average myofibrillar protein breakdown rate (Sandri, 2008). Our findings suggest that 3rd generation OCs containing gestoden or desogestrel influences net balance in response to exercise. To investigate molecular mechanisms underlying this effect, we analyzed intracellular signaling proteins involved in protein synthesis and degradation. One key regulator of muscle protein synthesis, mTOR increased in response to training in both groups. Furthermore, the E3 ligase MURF1 that recruits proteins for degradation in the proteasomes, tended to decrease only in the OC group, which is consistent with the observed greater muscle gain (*p* = 0.06) in OC than NOC. Although we did observe small changes in eIF4E and p70S6K, the remaining signaling proteins remained unchanged. Consequently, with this study, the changes in muscle mass does not seem to correlate with consistent changes in the investigated intracellular signaling proteins. However, the present study collected only resting biopsies and as such was not designed to investigate molecular mechanisms; thus, future studies should attempt to investigate the acute effect of OCs in relation to exercise training.

The included OC users had been using OCs for at least 3 months prior to the training period. Therefore, the latter findings suggest that use of OCs has a positive synergetic effect on resistance training response at the myocellular level. The activation of skeletal muscle signaling pathways by OCs when combined with regular training may involve activation of the estrogen receptors within the skeletal muscle. In support of this notion, human data have shown upregulation of myogenic-related gene expression after exercise when estrogen receptor α is activated (Haines et al., 2018). Our previous findings showed lower myofibrillar synthesis rate compared to control in users of OCs containing gestoden, but not levonorgestrel (Hansen et al., 2011), while the study by Ruzic et al. (2003) showed a greater increase in muscle strength and mass in levonorgestrel-users. These results suggest that the 3rd generation OCs containing low-androgen progestin (gestoden or desogestrel) reduces the muscle hypertrophic effect of resistance training compared to more androgenic types of OCs such as levonorgestrel. In the present trial, we only included users of 3rd generation OCs, but with a variable content of EE. *Post hoc* analysis of our data indicates the possibility that the potentiated hypertrophic effect of training and OCs on muscle mass may be related to EE concentration of the applied OC. This opens the possibility that EE in the OCs plays a direct stimulatory role when combined with regular resistance training. Still, it is unknown how such a stimulatory effect would be mediated. Previous studies utilizing postmenopausal women as an estrogen depleted-model, showed no effect of estrogen on myofibrillar FSR (Smith et al., 2014), however, the combined effect of estrogen supplementation and exercise increased myofibrillar FSR compared to controls (Hansen et al., 2012) implying an enhanced stimulating effect of estrogen on the exercise induced anabolic stimuli, which supports the current findings. We speculate that while endogenous estrogen levels fluctuate during the menstrual cycle, monophasic OCs provide an exogenous steady state level of EE introducing a continuous stimulus on the intramuscular ERs during day 1–21 of the OC cycle, which could provide the enhanced anabolic stimulus. Furthermore, EE is more potent than the endogenously produced 17β-estradiol in inducing estrogenic effects in women (Coelingh Bennink, 2004). Therefore, EE may influence myogenic gene expression directly as suggested by the present findings.

Another way OCs may influence the response to resistance training is by increasing recovery time (Minahan et al., 2015). It can be speculated that when exercising regularly, a delayed recovery between exercise sessions may induce a greater stimulatory response on muscle hypertrophy, but this statement needs to be investigated in future studies. Other factors that could potentially influence muscle mass in OC users are related to the changes in the endogenous hormone profile. Higher resting and exercise-induced GH levels have previously been observed in OC users compared to NOC users (Kraemer et al., 2008), while testosterone and IGF-1 levels have found to be lowered by OC (Hansen et al., 2009b). The effect of GH and IGF-I on muscle growth in humans is not clear (Heinemeier et al., 2012), but GH and IGF-I likely have a stimulatory effect on protein synthesis in the muscle connective tissue, ligaments and tendon, rather than stimulating myofibrillar protein synthesis (Doessing et al., 2010a) rate and muscle mass itself (West et al., 2009). However, it cannot be ruled out that a higher GH level in OC users may have had a positive regulatory influence on satellite cells, since the numbers of satellite cells increase in human muscle with increased GH/IGF-I levels (Doessing et al., 2010a,b). In the current study, serum testosterone was lower in OC users, whereas SHBG was higher compared to NOC users. This suggests that the level of free testosterone is also reduced, which is a well-documented observation (Zimmerman et al., 2014). Since testosterone has a stimulatory effect on muscle protein synthesis (Smith et al., 2012), the lower level of free testosterone in the OC users is not the explanatory factor for the enhanced muscle gain in the OC-users.

Likewise, progesterone is lower in OC users, which was also documented in the present study. The impact of progesterone on muscle mass is uncertain. Smith et al. found that skeletal muscle protein synthesis rate in post-menopausal women increases after progesterone administration (Smith et al., 2014). This could suggest an anabolic effect of progesterone. However, progesterone has also been proposed to exert a catabolic effects on skeletal muscle based on a higher lysine requirement observed in women during the luteal phase (Kriengsinyos et al., 2004), and greater urinary urea N excretion and total urea N excretion in sweat and urine in the ML phase compared to menses (Lamont et al., 1987), but data regarding resistance exercise are missing (Oosthuyse and Bosch, 2010). If progesterone has a catabolic effect on skeletal muscle mass, the lower level of progesterone in OC users could partly explain the greater muscle gain. Nevertheless, OCs also contain a synthetic form of progesterone (gestoden or desogestrel). The isolated effect of the progestin cannot be elucidated from the present *in vivo* study as only the combined effect OC containing both EE and progestin was investigated.

Resistance training aimed at increasing muscle hypertrophy and muscle strength as in the present study, generally increase CSA of the fast type II muscle fibers (Andersen and Aagaard, 2000). Accordingly, we observed non-significant increases in type II muscle fiber CSA in both OC and NOC and a shift from type IIx to type IIa muscle fibers. The decrease in type IIx underline that the resistance training program was effective in this group of previous untrained women. To our surprise, the CSA of the slow type I muscle fibers increased, but this was found in the OC users only. This observation for type I muscle fibers is supported by a study reporting a larger effect of phytoestrogen containing soy proteins on type I CSA as compared to whey protein or placebo (Haun et al., 2018). In line with this, testosterone supplement in strength training males seems to increase the growth of both type I and type II muscle fibers, whereas strength training alone is documented to primarily target type II fibers (Kadi et al., 1999; Kadi, 2000; Eriksson et al., 2005). In addition, it has been shown that type I muscle fibers have an enhanced sensitivity to hormonal anabolic stimuli (Hartgens et al., 1996), suggesting similar actions of the endogenous male and female sex hormones testosterone and estrogen directed is able to target both type I and type II muscle fibers and amplify the anabolic effect of strength training. In support of this, ER-β subtype selective agonist treatment in ovariectomized Wistar rats led to increased expression of Pax7 mRNA in the slow soleus muscle and increased muscle fiber size of type I muscle fibers (Weigt et al., 2012). Collectively, these observations may indicate that the EE component in OC positively influence the satellite cells associated with type I muscle fibers and thereby support an increased anabolic stimuli of OC, when combined with strength training. Nevertheless, further human studies are needed to confirm these findings.

The increase in muscle strength was not different between OC and NOC users (1RM 18 vs. 15%, MVIC 17 vs. 14%) even though the muscle hypertrophy tended to be greater in OC than NOC users (11 vs. 8%, interaction *p* = 0.06). Several factors may help to explain this observation. Neural and neuromuscular factors influence muscle strength (Moritani and deVries, 1979), as well as the levels of endogenous and synthetic female hormones may influence strength performance acutely on the day of testing (Sung et al., 2014), as indicated by a study in rodents showing a positive influence of estrogen on myosin structure and contractility (Lai et al., 2016). Variation in endogenous and exogenous female hormones between test-days may have introduced noise and thereby hindered the possibility of detecting differences in muscle strength. A prolonged training period and a greater samples size would have enhanced the possibility to detect a difference in muscle strength parameters between groups. Even small improvements in muscle mass and strength adaptations during a training period induced by OC use would be of great importance for e.g., elite athletes, but probably not be of clinical relevance during daily life activities in the general female population.

### Tendon Cross Sectional Area and Cross Links

There are very few studies investigating the role of female sex hormones on tendon tissue, yet, previous studies have indicated a lower tendon collagen FSR in women compared to men at rest and in response to exercise (Miller et al., 2006, 2007). Furthermore, Westh et al. (2008) did not detect any difference in Achilles and Patellar tendon CSA between experienced female runners and sedentary controls, whereas a greater tendon CSA was observed in the male runners after adjustment for body weight (Westh et al., 2008). In support, 9 month of running (in total 43 h) lead to a 9% increase in VO_2max_, but no change in Achilles tendon CSA (Hansen et al., 2003). These observations indicate a reduced ability to increase tendon CSA in female athletes compared to male athletes. Nevertheless, in the present study we observed an increase in tendon CSA in the mid portion of the patella tendon in both OC and NOC users, indicating that women show tendon adaptations to resistance training interventions. In support, female handball players show a trend toward a greater tendon CSA, when comparing the preferred jumping leg with the contralateral leg (*P* = 0.09) (Hansen et al., 2013). These observations are in contrast to the observation by Westh et al. (2008). An explanation for the discrepancy may be that they included female runners, in whom, a low energy intake and disturbance in hormonal profile may have hampered their ability to adapt (Farup et al., 2014; Maughan et al., 2018). Another explanation might be that the loading threshold for a hypertrophic effect of regular mechanical loading is higher in women than in men and the stimulating effect of running may be below that threshold.

Both OC and NOC increased tendon CSA. Previous observations showed a lower tendon collagen synthesis rate in OC compared to a NOC group at rest (Hansen et al., 2009b) and in response to exercise (Hansen et al., 2008), which was speculated to be caused by a lower bioavailability of IGF-I locally in the tissue and blood in OC-users (Maughan et al., 2018). In support of a slower tendon collagen turnover in OC-users, we in the present study observed a tendency toward a greater content of HP compared to NOC (group effect, *p* = 0.07) (Hansen and Kjaer, 2016). Based on this, we expected to see a hampered response to training in tendon CSA in the OC users compared to NOC. On the other hand, if OC enhances the gain in muscle mass as suggested by the present findings, this may increase the loading on tendon and ligaments during maximal locomotion and thereby enhance the stimulatory effect of exercise on tendon adaptation. In support, cross-sectional data from experienced female handball players show greater tendon CSA adjusted for body weight (mm^2^/kg^2/3^) of the jumping leg compared with the contralateral leg at the distal level of the patellar tendon in OC users, but not in NOC users (*p* = 0.98, interaction *p* = 0.08) (Hansen et al., 2013).

In the present study, we did not observe any increase in CSA neither in the distal nor in the proximal parts of the tendon in the OC group, while the NOC users increased CSA in all three parts of the tendon. On top of a higher content of tendon HP cross-links supports the notion of a slower collagen FSR in users of OC compared to NOC (Hansen et al., 2008). In addition, the improved gain in muscle mass in OC users compared to NOC, along with a comparable increase in tendon CSA, may indicate that OC users need more time to adapt in tendon than in the contractile muscle pointing at a mismatch in adaptation between tissues and an increased risk of injury. Further elucidation of this aspect is needed, but the observation should be kept in mind when female athletes using OCs are adapting to new or increased training loads.

### Methodological Considerations

Use of OC or NOC was not randomized in our study, and non-training groups would be required to clarify the entire effect of OC-initiation on muscle mass and strength. On the other hand, a strength of the present study where we included habitual users of OC, is that they have bypassed any transient changes taking place in the first months after initiation of OC use, such as changes in endogenous hormones (e.g., lower endogenous estradiol and testosterone levels and increasing SHBG levels) and mental side-effects (Kuhnz et al., 1993; Skovlund et al., 2016). If OC use was initiated concurrent with the exercise intervention, we would be unable to separate the effects of the two.

We did not control for the time-point in menstrual cycle phase/pill cycle when testing muscle strength. This was done so, as our primary outcome parameter in the study was potential improvements in skeletal muscle mass with training, and thus, we aimed for an equal number of training sessions between individuals of the groups. Should the menstrual cycle have been taken into account, it would have been necessary to include a different number of exercise sessions based on differences in the length of the menstrual cycle (e.g., 25 vs. 35 days) or changed the recovery length between exercise sessions. Both adjustments might have affected the results in regards to muscle mass gain, and thus, conflicted with the primary goal of the study. Nevertheless, the lack of control for the menstrual cycle time-point may have introduced variations during our strength testing. In the NOC users, the progesterone level was higher in 8/13 women when testing after the training period compared to baseline, whereas it was lower only in 2/13 and comparable in the last 3/13 subjects. This suggests that a greater number of NOC users were within the luteal phase of menstrual cycle when testing after the intervention compared to before the intervention. The literature within this area report conflicting data. Some studies have found a greater maximum voluntary force production around the time of ovulation (Phillips et al., 1996; Sarwar et al., 1996; Iwamoto et al., 2002), while others report no variation in strength parameters between menstrual phases (Constantini et al., 2005; Lebrun et al., 2013). Yet, such variations in performance may have weakened our possibility for detecting a difference in strength gain between the study groups. The training period was 10 weeks in the present trial. The results from a few training studies indicate that the gain in muscle mass and strength is greater when the training sessions are concentrated in the follicular phase rather than in the luteal phase or equal distributed during the cycle (Reis et al., 1995; Sung et al., 2014; Wikstrom-Frisen et al., 2017). However, others have reported no difference in adaptation to resistance training related to the timing of the training sessions in different menstrual phases (Sakamaki-Sunaga et al., 2016). In light of an average 28-day menstrual cycle, it would in theory have been optimal, if the training period was 12 weeks corresponding to three average cycles. The training sessions were equally distributed each week during the training period; thus, we cannot rule out that some participants have performed a larger part of their training within the follicular phase, related to timing of the menstrual cycle. As discussed previously, it is difficult to control the latter.

The participants collected 4-day dietary records. The results indicated a rather low energy intake considering that the subject are young and exercising three times per week, while also remaining weight stable throughout the 10-week intervention. This suggest that the two 4-day dietary records are not representative of the total amount of energy really consumed during the 10 weeks of training.

The dietary records also indicated a lower protein intake per kg body weight in the NOC users compared to the OC users (Table 5). This could potentially have lowered the ability of the NOC users to synthesize new myofibrillar protein and thereby gain muscle mass. However, we observed no correlation between muscle gain measured by MRI and protein intake in the participants (OC *r* = 0.045, *p* = 0.88; NOC *r* = 0.37, *p* = 0.24) (Supplementary Figure S4). This indicates that the reported lower protein intake in NOC may not have influence the gain in muscle mass in the present study. It is worth noticing that self-reported dietary intake is subject to misreporting (Bokhof et al., 2010), which question the validity of self-recorded food records in general. In any case, to ensure an equal availability of amino acids for myofibrillar protein synthesis in the hours after each exercise session, the participants in both groups ingested a standardized protein bar immediately after every training session (Andersen et al., 2005).

## Conclusion

The present results suggest that habitual users of monophasic 3rd generation OCs experience a tendency toward a potentiated anabolic effect of resistance training on skeletal muscle mass compared to NOC. Moreover, we demonstrate that untrained women can increase patellar tendon size in response to regular resistance training irrespective of OC use. Despite the trend toward altered muscle adaptation to resistance training with the use of OC, this did not significantly influence the muscle strength improvement following strength training in young women.

## Data Availability

All datasets generated for this study are included in the manuscript and/or the Supplementary Files.

## Ethics Statement

All subjects gave written informed consent in accordance with the Declaration of Helsinki. The protocol was approved by the Central Denmark Region Committee on Health Research Ethics (Journal No.: M-20100187).

## Author Contributions

HS-J, JJ, CC, HL, MK, and MH conceived and designed the study. UD, NR, and MH carried out the experiments. LD, UD, JA, AM, VK, and MH analyzed the data. LD, UD, JA, AM, VK, and MH interpreted the results of the experiments. LD and MH prepared the figures. LD and MH drafted the manuscript. LD, UD, JA, NR, AM, HS-J, JJ, VK, CC, HL, MK, and MH edited and revised the manuscript. LD, UD, JA, NR, AM, HS-J, JJ, VK, CC, HL, MK, and MH approved the final version of the manuscript.

## Conflict of Interest Statement

The authors declare that the research was conducted in the absence of any commercial or financial relationships that could be construed as a potential conflict of interest.
