# Supplementary material for: Influence of Oral Contraceptive Use on Adaptations to Resistance Training
Source: Front Physiol. 2019 Jul 2;10:824. doi: 10.3389/fphys.2019.00824 (PMC6614284; doi:10.3389/fphys.2019.00824)
Supplement: Supplementary file 1 [file Data_Sheet_1.PDF]

Supplemental material

Influence of oral contraceptive use on adaptations to resistance training

Line B. Dalgaard[1], Ulrik Dalgas[1], Jesper L. Andersen[2], Nicklas B. Rossen[3,4], Andreas Buch Møller[3], Hans Stødkilde-Jørgensen[5], Jens Otto Jørgensen[3,4], Vuokko Kovanen[6], Christian Couppé[2], Henning Langberg[2,7], Michael Kjær[2], Mette Hansen[1,2]

[1] *Section of Sport Science, Department of Public Health, Aarhus University, Aarhus, Denmark.*
[2] *Institute of Sports Medicine, Dept. Orthopedic Surgery M, Bispebjerg Hospital and Center for Healthy Aging, Faculty of Health Sciences, University of Copenhagen, Copenhagen, Denmark.*
[3] *Steno Diabetes Center Aarhus, Aarhus University Hospital, Aarhus, Denmark.*
[4] *Department of Endocrinology and Internal Medicine, Medical Research Laboratories, Aarhus University Hospital, Aarhus, Denmark.*
[5] *Center of Magnetic Resonance, Aarhus University Hospital, Skejby, Denmark.*
[6] *Faculty of Health Sciences, University of Jyväskylä, Jyväskylä, Finland*
[7] *CopenRehab, Department of Public Health, Faculty of Health and Medical Sciences, University of Copenhagen*

Supplemental Figure S1

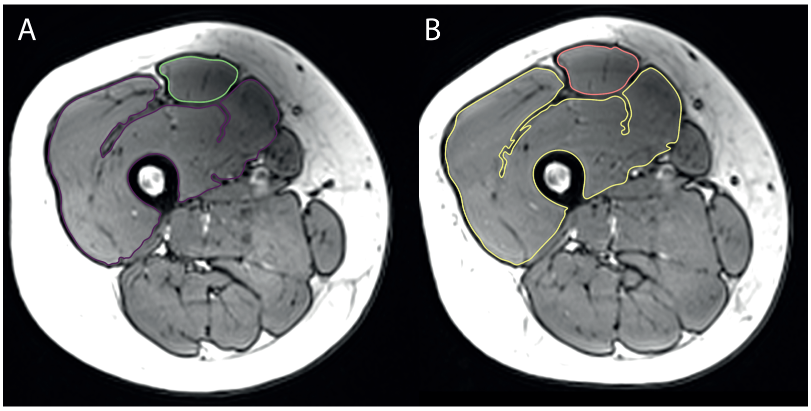

Figure S1: Representive images of MRI scans of baseline (A) and post (B) muscle CSA at 20 cm. The colored outline illustrates the procedure of measuring the CSA. A mean value of three measurements of the same image was included in the analysis.

Supplemental Figure S2

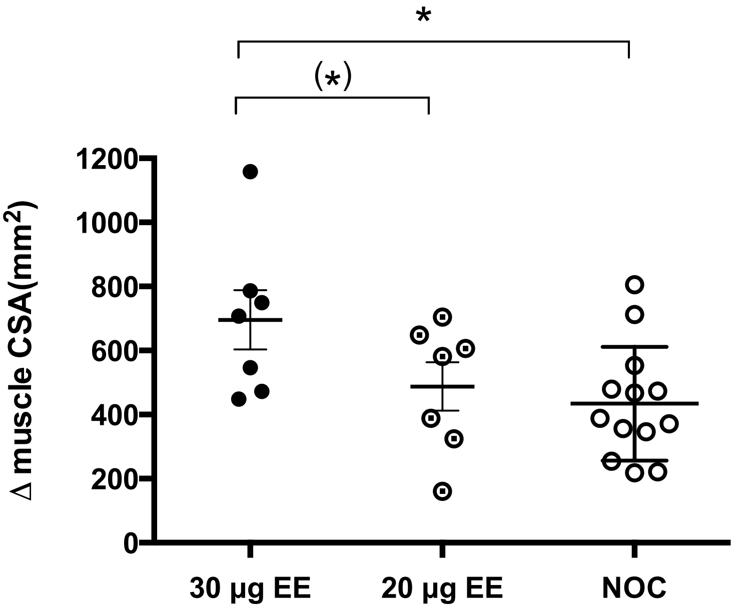

Figure S2: Changes in muscle CSA in users of oral contraceptives (OCs) with 30 μg EE (n=7), 20 μg EE (n=7) or NOC (n=13). 20 μg EE vs. 30 μg EE: p=0.08. 30 μg EE vs. NOC: p=0.01. 20 μg EE vs. NOC: p=0.73. NOC: non-oral contraceptives users. Black circles: 30 μg EE. Partly filled cicles: 20 μg EE. Open circles: NOC.

Supplemental Figure S3

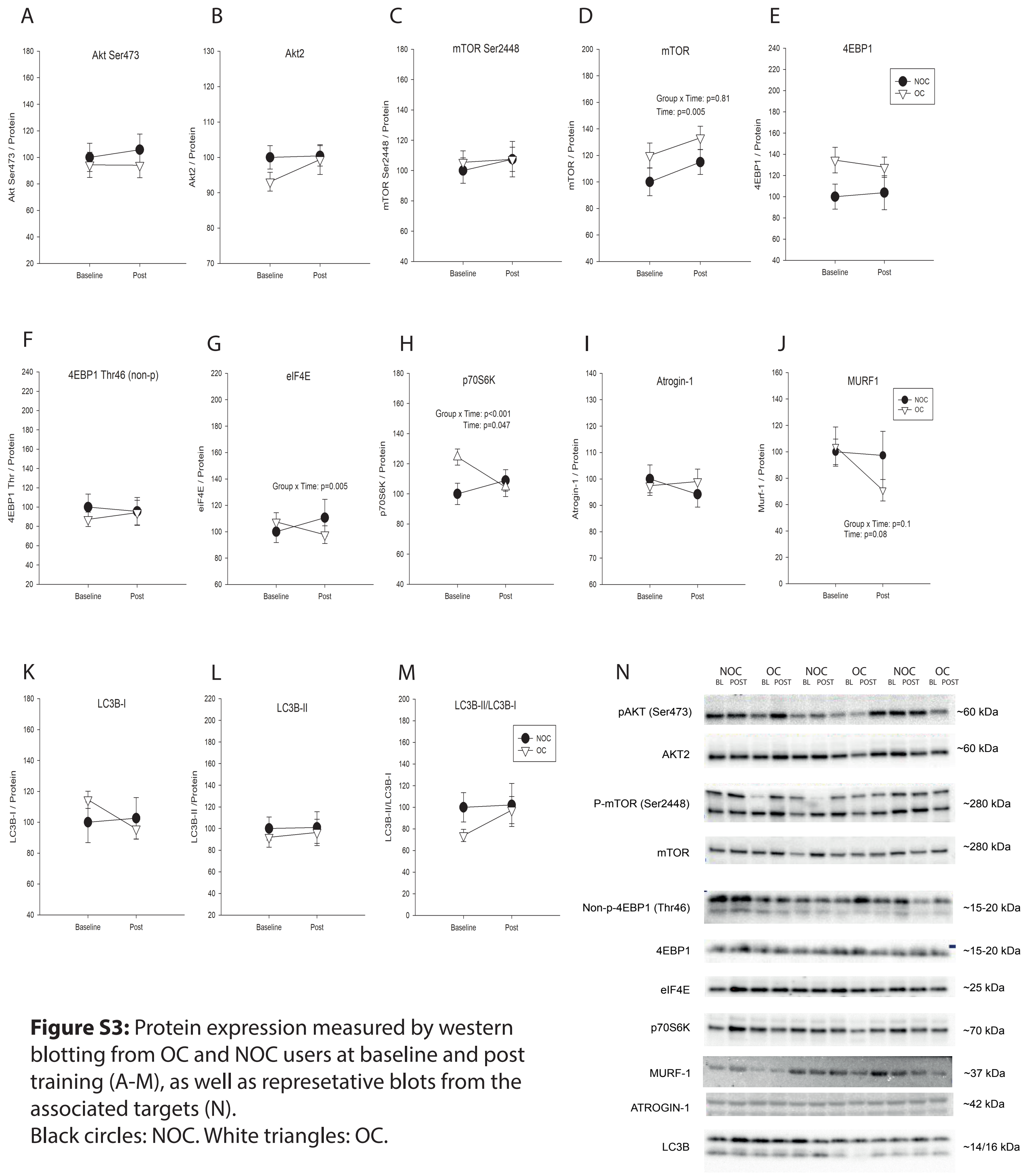

Figure S3: Protein expression measured by western blotting from OC and NOC users at baseline and post training (A-M), as well as represetative blots from the associated targets (N).
Black circles: NOC. White triangles: OC.

Supplemental Figure S4

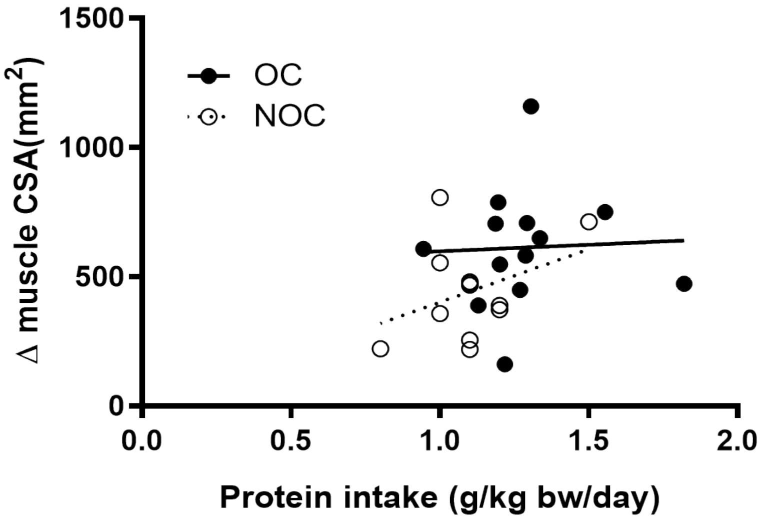

Figure S4: Correlation between daily protein intake per kg body weight (g/kg bw/day) and muscle mass gain determined by MRI in OC and NOC users. OC: Filled circles, p=0.88, r=0.045. NOC: Open circles, p=0.24, r=0.37.
